# Public Opinions on Using Social Media Content to Identify Users With Depression and Target Mental Health Care Advertising: Mixed Methods Survey

**DOI:** 10.2196/12942

**Published:** 2019-11-13

**Authors:** Elizabeth Ford, Keegan Curlewis, Akkapon Wongkoblap, Vasa Curcin

**Affiliations:** 1 Department of Primary Care and Public Health Brighton and Sussex Medical School Brighton United Kingdom; 2 Department of Informatics King's College London London United Kingdom; 3 School of Population, Health and Environmental Sciences Faculty of Life Sciences and Medicine King's College London London United Kingdom

**Keywords:** social media, depression, mental health, machine learning, public opinion, social license, survey

## Abstract

**Background:**

Depression is a common disorder that still remains underdiagnosed and undertreated in the UK National Health Service. Charities and voluntary organizations offer mental health services, but they are still struggling to promote these services to the individuals who need them. By analyzing social media (SM) content using machine learning techniques, it may be possible to identify which SM users are currently experiencing low mood, thus enabling the targeted advertising of mental health services to the individuals who would benefit from them.

**Objective:**

This study aimed to understand SM users’ opinions of analysis of SM content for depression and targeted advertising on SM for mental health services.

**Methods:**

A Web-based, mixed methods, cross-sectional survey was administered to SM users aged 16 years or older within the United Kingdom. It asked participants about their demographics, their usage of SM, and their history of depression and presented structured and open-ended questions on views of SM content being analyzed for depression and views on receiving targeted advertising for mental health services.

**Results:**

A total of 183 participants completed the survey, and 114 (62.3%) of them had previously experienced depression. Participants indicated that they posted less during low moods, and they believed that their SM content would not reflect their depression. They could see the possible benefits of identifying depression from SM content but did not believe that the risks to privacy outweighed these benefits. A majority of the participants would not provide consent for such analysis to be conducted on their data and considered it to be intrusive and exposing.

**Conclusions:**

In a climate of distrust of SM platforms’ usage of personal data, participants in this survey did not perceive that the benefits of targeting advertisements for mental health services to individuals analyzed as having depression would outweigh the risks to privacy. Future work in this area should proceed with caution and should engage stakeholders at all stages to maximize the transparency and trustworthiness of such research endeavors.

## Introduction

### Depression

At any given time, 1 in 6 adults (17%) in Western high-income countries such as England experience a common mental disorder (CMD) such as depression [1]. Depression, alongside other CMDs, accounts for nearly half of all ill health in people younger than 65 years [2,3]. It has a notable impact on both individuals and society; 90% of people who die by suicide have a mental health condition at the time of their death, and the highest rates of suicide are associated with depressive disorders [4]. Depression is also associated with a loss of productivity [5]. Mental illness in England is thought to cost the economy £105.2 billion each year due to factors such as time off work, reduced quality of life, and costs of running services [6]. However, only 1 in 3 adults (37%) aged 16-74 years with depression currently get access to mental health treatment [1].

### Underdiagnosis and Undertreatment of Depression

In the United Kingdom, patients usually first seek National Health Service (NHS) health care through their general practitioners (GP) who manage up to 90% of mental health consultations [7]. People with mental health problems can alternatively seek specialized help directly from both NHS services, such as Improving Access to Psychological Therapies and charities. However, there is a geographical variance in the availability of services between regions, and there is still a significant proportion of the UK population who are underdiagnosed and undertreated [8].

Research shows that depression is underdiagnosed in general practice, with less than half of likely cases being recorded in patient notes [9-11]. The diagnosis of depression can be challenging, with some patients presenting with undefined or somatic illness [12]. Those who suffer from depression additionally may not disclose their symptoms to their GP, with common reasons including fear of stigmatization, concerns about privacy regarding medical records being seen by employers, and medication aversion [13]. Even if depression is diagnosed correctly in primary care, it is often undertreated because of the lack of service accessibility and long waiting times. Over 12% of people wait longer than 1 year in the United Kingdom to start nonpharmacological treatment, and 54% of people wait over 3 months [14]. This is in part due to the reductions in the availability of resources dedicated to mental health care as well as the increase in demand for these services, which contributes to the long waiting lists [14]. The impact on patients’ quality of life from the underdiagnosis of depression may be considerable, given that mental illness has the same effect on life expectancy as smoking, [2].

Current evidence, therefore, suggests that the needs of patients with depression in the United Kingdom are not being met within the NHS. The gap between diagnosis and treatment of depression could be bridged by charities and third sector organizations, which provide services and treatment to eligible individuals. However, these services are not always publicized widely.

### Why Use Social Media?

Social media (SM) offers a promising avenue for targeting information about third sector mental health services to people who need them. SM sites such as Facebook already use algorithms to target advertisement to the most appropriate users, for example, by using search keywords from the history of search engines and links that users have previously clicked on. As machine learning and other computer science techniques have become more advanced, it is increasingly possible to identify or predict specific characteristics, such as mood or depression, of SM users, from the content they post on sites such as Facebook or Twitter [15-18]. This may involve sentiment analysis (the valence of the emotion or mood of their words), analyzing posted images, or recognizing changes in the quantity and frequency of a user’s content [19]. Previous research has shown that users disclose depressive symptoms on SM sites such as Facebook [20] and Twitter [21]; in some cases, users disclose enough information for researchers to make a diagnosis of a major depressive episode [20].

Thus, an algorithm could be developed that would identify Facebook users who are experiencing low mood or depression [22]. A mental health charity could then use this algorithm to selectively target the advertisements for its services to the most likely users. Alternatively, pharmaceutical companies could use such technology to target their drugs to the appropriate patient population.

### Ethical Issues With Using Machine Learning to Target Advertising for Depression Services

This targeted advertising of mental health services would operate with the intention of promoting help to those who need it, as opposed to marketing goods for financial gain. However, some users of SM may find the notion of profiling their content for their mental health status in a SM forum as unacceptably intrusive. Privacy has been identified as an important concern for population-level SM research, with the association of individuals with a potentially stigmatizing medical condition being an established worry of users [23]. The possibility of breaches in confidentiality, stigmatization, and the consequent modification in SM use due to awareness of this profiling (*known as the Panoptican effect*) are corollaries of such SM analysis, which may cause the public to view it negatively [24]. Chancellor et al established a broad taxonomy of ethical tensions in inferring mental health states from SM, grouping them into issues around ethics committees and the gap of SM research; questions of validity, data, and machine learning; and implications of SM research for key stakeholders [25].

### Aims of This Study

Considering the reasons outlined above, the public may have stronger views on the use of targeted advertising for mental health services than for other goods or services. We believe that it is important to understand what SM users think of algorithms for identifying depression from SM content, which would be used for target advertising. Specifically, we seek to understand whether the public is in favor of the analysis of SM content for possible mental health problems.

In this study, members of the public and users of mental health charity services completed an online questionnaire, which aimed to find out the following:

1. Whether SM users feel their posted content reflects their mental health reality.
2. If SM users are largely in favor of:
  - SM content being analyzed for indications of mental health problems and
  - SM content being used to guide targeting of advertising about mental health services.
3. Which aspects of analyzing or targeted advertising make people feel comfortable or uneasy.
4. Whether there are differences of opinion by demographic group.
5. Qualitative reactions to the topic.

## Methods

### Ethics Statement

This study was reviewed by and received favorable ethical opinion from the Brighton and Sussex Medical School Research Governance and Ethics Committee (*ref ER/BSMS2730/1*).

### Study Design

This study is an online open cross-sectional survey designed on Qualtrics.

### Participants

Any SM user within the United Kingdom was eligible to complete the survey, with no restrictions on eligibility except that respondents should be aged 16 years or older.

### Questionnaire

The questionnaire was developed by authors EF and KC, with comments and suggestions made by all authors. It was developed using an iterative process of item generation, discussion among all authors, and refinement, based on general themes and participant quotes within the relevant literature [17,23,26]. To meet all 5 research objectives, we created questions on the following topics: (1) participant characteristics, (2) participants’ SM usage, (3) participants’ experience of depression and views on how this influences their SM use, (4) views on the analysis of SM content for depression and targeted advertising for mental health services, and (5) whether participants would support the use of algorithms for identifying depression from their SM content.

The full questionnaire is provided in Multimedia Appendix 1. Questions 1 to 5 captured the demographics and SM usage of the participants. Questions 6 to 11 were simple closed questions that asked the participants how their mood may affect their use of SM. Question 11 and 12 focused on the use of SM and attitudes to privacy. Questions 13 to 15 were generated following review of previous literature on the same topic [17,23,26]. Before these questions, which asked for views on analyzing SM content for targeted advertising, some simple explanatory information was provided about the technical aspects of targeted advertising to ensure that the participants were able to answer these questions. In question 15, we focused and adapted the themes raised within the literature to generate statements with Likert scale responses, which presented a range of possible reactions to targeted advertising for depression services, with which participants could agree or disagree. We also included open-ended questions (16 to 18) to capture the themes that we may not have addressed within the structured questions. The questionnaire contained a range of multiple-choice questions, matrix tables, and free-text boxes to ensure that a range of both quantitative and qualitative data was generated from the participants across all aspects of the above-mentioned 5 research objectives.

### Recruitment and Procedure

The survey was widely advertised on mental health charity websites and SM pages, including Mind, Turning Point, Samaritans, and MQ mental health. It was actively promoted through Facebook, Twitter, and Instagram via paid advertisements and using personal and institutional accounts. Local community groups and mental health support groups on SM sites were also asked to promote the questionnaire. It was disseminated through mailing lists, such as through Brighton and Sussex Universities, and through medical informatics communities, such as the Farr Institute, especially to public panels and interest groups. Due to this method of advertising, it was not possible to estimate response rates to the advertisement for the study. Full study information appeared on the first page of the questionnaire website. Participants were asked to indicate that they had read and understood the information and wished to provide their consent by clicking on a box. Then, the participants completed the questionnaire in their own time. Recruitment was open from February 1, 2018, to October 24, 2018.

### Data Analysis

Data were downloaded from Qualtrics into IBM SPSS Statistics version 25.

Quantitative analysis was conducted by the calculation of summary statistics using frequencies and averages. Gender and ethnicity were dichotomized (into male and female, and white and nonwhite, respectively), and comparisons for these variables, as well as previous depression status, were made using a chi-square test.

Free-text answers were downloaded into NVivo version 12 (QSR International Pty Ltd), and qualitative analysis was performed by author KC using thematic analysis according to the 6 phases defined by Braun and Clarke [27]. This was a recursive process, which involved the coding of participants’ responses using NVivo and the creation of multiple thematic maps. Codes were aggregated into meaningful groups, and a minimum number of meaningful themes emerged from the data, which best represented the common topics in participants’ responses in the most parsimonious way.

## Results

### Participant Characteristics

In total, 183 full responses were recorded, with participant characteristics shown in Table 1. Participants were spread fairly evenly across age groups from 16 to 65 years, with 1 respondent aged over 65 years. There was a slight underrepresentation of the younger age groups when compared with the percentage of SM users across the United Kingdom [28]. Twice as many females undertook this survey as compared with males, which may be explained by more females using SM than males [29] and that depression is more prevalent among females [30]. In this sample, 85.3% of the participants were of white ethnicity, which is similar to the population of England and Wales where 86% of the residents are white [31].

### Use of Social Media

Facebook, Twitter, and Instagram were the SM platforms most used by participants. Facebook was the most frequented SM site (Table 2).

Participants mostly posted content on Facebook for only their friends to view. Posts regarding personal feelings or asking for support were posted publicly the least. Those who took the survey stated that their posts that were public were largely reserved for impersonal content such as advertising or sharing content from other sources (Table 3).

**Table 1:** Participant characteristics (N=183).

Characteristics		Values
Age (years), mean (SD)		38 (11.66)
**Age groups (years), n (%)**		
	16-24	24 (13.1)
	25-34	50 (27.3)
	35-44	51 (27.9)
	45-54	38 (20.8)
	55-64	19 (10.4)
	65+	1 (0.5)
**Gender, n (%)**		
	Female	127 (69.4)
	Male	54 (29.5)
	Other	2 (1.1)
**Ethnicity**		
	White British	111 (60.7)
	Any other white background	45 (24.6)
	White and black Caribbean	1 (0.5)
	White and black African	2 (1.1)
	White and Asian	1 (0.5)
	Indian	6 (3.3)
	Pakistani	2 (1.1)
	Chinese	6 (3.3)
	Any other Asian background	5 (2.7)
	Arab	3 (1.6)
	Any other ethnicity or background not stated	1 (0.5)

**Table 2:** Use of social media sites and frequency.

Social media sites	Participants using site, n (%)	Frequency of use (% of participants using site), n (%)			
		Many times a day	Once a day	A few times a week	Less than once a week
Facebook	159 (84.6)	103 (64.8)	28 (17.6)	16 (10.1)	12 (7.5)
Twitter	118 (62.8)	49 (41.5)	22 (18.6)	27 (22.9)	20 (16.9)
Tumblr	5 (2.7)	1 (20.0)	0 (0.0)	1 (20.0)	3 (60.0)
Instagram	99 (52.7)	47 (47.5)	20 (20.2)	15 (15.2)	17 (17.2)
Snapchat	44 (23.4)	13 (29.5)	7 (15.9)	6 (13.6)	18 (40.9)
Flickr	10 (5.3)	0 (0.0)	0 (0.0)	0 (0.0)	10 (100.0)
Other	18 (9.6)	2 (11.1)	4 (22.2)	3 (16.7)	9 (50.0)

**Table 3:** Expected audience for content posted on Facebook. Survey answers for question, "Thinking specifically about Facebook: please select the type of content you post".

Type of content posted on social media	Publicly, n (%)	To friends only, n (%)	To closed groups, n (%)	To open/interest groups, n (%)
Share articles/pictures/quotes from other sources	34 (18.6)	142 (77.6)	4 (2.2)	3 (1.6)
Describe my current life events/share my news	11 (6.0)	161 (88.0)	11 (6.0)	0 (0.0)
Describe my state of mind	21 (11.5)	138 (75.4)	21 (11.5)	3 (1.6)
Ask for advice or support	6 (3.3)	118 (64.5)	53 (29.0)	6 (3.3)
Advertise goods or services/seek goods or services	50 (27.3)	71 (38.8)	36 (19.7)	26 (14.2)
Other—if so, please be specific and tell us the type of content (eg, “Checking in,” tagging in memes, sharing own art or pictures, professional content, and sharing political content).	7 (43)	6 (38)	3 (19)	0 (0)

### Relationship Between Depression and Use of Social Media

Over half of the participants had experienced depressive symptoms that had made them consider seeking help (62.3% [114/183]; Table 4). This high figure is assumed to be a result of advertising via mental health charities, which gave us access to the above-average number of patients who had experienced depression. In total, 22.7% (40/176) of the participants agreed that their recent low mood would be evident from their SM activity, and most of the participants thought that posted SM content is not reflective of true feelings. As shown in Table 4, three-quarters of participants who answered the question (N=44) stated that they often post less on SM than usual when they are feeling low and only 11% (5/44) post specifically to seek support. Within the same group of participants (N=44), 70% (31/44) agreed that when they are feeling low, they do appreciate getting support from friends on SM.

**Table 4:** Relationship between depression and use of social media.

Question		Respondents who answered “Yes,” n (%)	Total number of respondents per question, n
Have you ever experienced depressive symptoms long or severe enough that you have thought about seeking help?		114 (62.3)	183
If you have experienced low mood in the recent past, do you think this would be evident from your online public social media activity?		40 (22.7)	176
Is your posted social media content reflective of your true state of mind when you are feeling low?		14 (3)	44
**How much do you tend to post when your mood is very low?**			
	More than usual	5 (11)	44
	Same as usual	6 (14)	44
	Less than usual	33 (75)	44
When you are feeling low, do you appreciate getting support from friends on social media?		31 (71)	44
Do you post on social media specifically to seek support for your low mood?		5 (11)	45

### Views and Perceptions Around the Profiling of Social Media for Mental Health

Participants responded to a series of statements using a 5-point Likert scale, with mean responses and standard deviations reported in Table 5 (higher scores equaled a positive agreement). Participants’ scores were largely toward the “disagree” end of the scale when they were asked if they would feel comfortable with their Facebook posts being analyzed for target advertising. This was regardless of the type of advertising, although advertising from brands and businesses was viewed least favorably.

As a whole, participants scored more toward the “agree” end of the scale when they were asked about the potential negative *and* positive impacts of the analysis of Facebook content for depression. The negative impacts included stigma, exposure, intrusiveness, and risk to privacy, whereas the more positive impacts included a widening access to services and reaching those who struggle to seek help. On balance, participants did not endorse the idea that the benefit to both individuals and society as a result of this analysis would outweigh the risk to individual privacy (Table 5).

In addition, participants felt uncomfortable with the idea of this analysis happening and felt least comfortable with the idea of a human analyzing their Facebook content for depression, compared with a computer algorithm.

A final question, with a yes/no response, was asked to the participants to ascertain if they supported this analysis and if they would be happy for their own data to be used in this way. In total, 60.0% (96/160) of the participants supported the idea of the use of software to analyze Facebook content for the purpose of improving targeting of charitable mental health care services. However, slightly less than half (43.9%, 69/157) of the participants would give consent for their own SM to be analyzed and even fewer (15.3%, 24/157) participants would be comfortable with this happening to their data without explicit consent (Table 6).

**Table 5:** Views on analyzing social media for targeting mental health services (5=strongly agree, 3=neutral, and 1=strongly disagree).

Question		Value, mean (SD)
**Would you feel comfortable if you discovered that posts on Facebook were being analyzed to target individuals for**		
	Advertising from brands and businesses	2.44 (1.10)
	Health care advice, for example, from the National Health Service	2.76 (1.32)
	Mental health care/advice	2.74 (1.34)
	Services offered by mental health charities, for example, Samaritans, Mind, or Turning Point	2.79 (1.32)
**How much do you agree with the following statements about analyzing Facebook users’ content for depression?**		
	It would increase stigmatisation.	3.10 (1.15)
	People might end up being outed as having depression.	3.73 (1.05)
	It would make me feel uneasy.	3.69 (1.21)
	I would find this intrusive.	3.80 (1.19)
	It would increase people’s access to mental health services.	3.34 (1.15)
	It could identify people who struggle to seek help in real life.	3.58 (1.02)
	I would be worried about my privacy if my Facebook was analysed in this way.	3.91 (1.14)
	The benefit to society outweighs the risk to my privacy.	2.73 (1.24)
	The benefit to individuals outweighs the risk to my privacy.	2.80 (1.26)
**I would feel comfortable if**		
	I knew this was happening.	2.66 (1.31)
	I knew a human was analysing my Facebook content for depression.	2.25 (1.22)
	A computer algorithm (not a human) was analysing my Facebook content for depression.	2.68 (1.35)

**Table 6:** Personal views on the use of own social media data for analyzing for depression. Survey answers to the question, "It may be possible using computer programming software, to work out from Facebook content whether a user is depressed or experiencing low mood to provide information about services that may be available. If this technique is shown to work well:".

Question	Participants who answered “Yes,” n (%)	Total number of respondents per question, n
In general, I support the idea of the use of this software.	96 (60.0)	160
I would give consent for my Facebook content to be analysed for depression.	69 (43.9)	157
I would be comfortable with my Facebook content being analysed for depression without my explicit consent.	24 (15.3)	157

### Differences of Opinion by Demographic Group

The proportions of participants responding positively to the final question were examined by age group, gender, ethnicity, and previous depression status (Table 7). In general, the younger age groups were more supportive of the use of this technology and were more willing to give consent for their own Facebook to be analyzed, although the age group of 55- to 64-year-olds was the group most supportive of this analysis being conducted without explicit consent. In particular, the age group of 16- to 24-year-olds was particularly supportive of this software if they could give their consent to its use, and the age group of 35- to 44-year-olds was the least supportive overall (not examined for statistical significance). A Pearson chi-square test was conducted to determine whether there was a difference in opinion for gender (male and female), ethnicity (white and nonwhite), and previous depression across 3 questions assessing support for the software, willingness to give consent, and whether users felt comfortable with the analysis happening without their consent. No significant results were found.

**Table 7:** Differences of opinion by demographic group.

Demographics		Percentage of those who support the idea of the use of this software	Percentage of those who would give consent for their Facebook content to be analyzed for depression	Percentage of those who would feel comfortable without explicit consent
**Age group (years)**				
	16-24	85	70	20
	25-34	65.1	51.2	22.2
	35-44	52.3	31.8	11.4
	45-54	60	40	8.57
	55-64	38.9	35.3	25
	65+	100	100	0
**Gender**				
	Female	58.4	41.1	14.3
	Male	63	50	15.9
	Other	100	100	100
**Ethnicity**				
	White background	59.1	44.8	14.9
	Nonwhite background	65.2	39.1	17.4
**Experiences of depression**				
	Previous depression	58.3	41.2	15.7
	No previous depression	65.6	52.5	15.3

### Reasons for Responses

Participants’ responses to the open-ended questions varied with strong views expressed regarding both the positive and negative aspects of the use of the software. Thematic analysis resulted in 3 themes describing the perceived benefits of the analysis (improvement of services, improvement of diagnosis, and societal benefit) and 3 themes describing concerns (privacy, usefulness, and accuracy of the software).

#### Benefits

##### Improvement of Current Mental Health Services by Increasing Access to Resources

A recurring theme was that the use of this software could assist in improving access to mental health services for those who needed them.

Participants mentioned that by providing targeted advertisement, the technology could increase the awareness of services available and, therefore, access to them:

If people with depression occasionally got targeted ads for e.g. CBT or other therapies they might be more inclined to have a go and potentially seek more help to get better.

People would be made aware of services available to them. They might realise the difficulties they are facing.

Participants also recognized that provision of resources could be improved by the software through the use of demographic analysis. This could increase access to services by ensuring that the services that are available are appropriate for different members of society:

Considered at population level, it could provide an overview of the depression and anxiety at a population level, and could be broken down demographically too. This could help provision of resources.

##### Improvement of Diagnosis

Another key benefit that was raised by the participants was that the software would help improve the diagnosis of depression, which is vital given that many are undiagnosed for multiple reasons and, therefore, cannot get access to treatment and support [32]:

I think it could help identify people with mild to moderate depression who are not aware that this is the cause of them struggling with life to offer them support that could improve their wellbeing and quality of life.

Participants suggested that the software would be of particular use in diagnosing those that the system currently misses. Participants recognized that the users of SM may find it more comfortable to post about their feelings than speaking about them in real life, and this could be of use in improving the rates of undiagnosed depression:

It could help out people who are more introverted and may not speak to other people about how they are feeling.

It is easy to have depression without identifying it as such. Increased opportunities for diagnosis are therefore a good thing.

It would be beneficial if it made it easier for people who are struggling had easier access to people who could help them in real life.

##### Societal Benefits From Advertising

Participants also recognized that targeting advertising already occurs, and some of the participants stated that the use of targeted advertising for the provision of mental health care was preferable to its current use:

Better than what it's currently used for…

None - we are all being targeted anyway with everything else, great idea.

#### Concerns

##### Privacy

Privacy was a key concern that was identified by a significant majority of respondents. Of particular concern was the potential for the data that were harvested to be exposed to others with untrustworthy motives. Stigmatization and discrimination were explicitly mentioned as worries:

With the number of data leaks we have by large tech companies, this is a risk too far for many people.

I don’t want people to be profiled, as social media is also a platform for self-expression. This could be used to discriminate against people for health and insurance reasons if the information were identifiable.

They’ll sell the information to anyone, Facebook only exists to make money out of people. This sort of analysis will probably be sold or hacked and would be detrimental, e.g. upsetting the individual and affecting things such as insurance, credit ratings etc.

In light of recent revelations about the questionable ethics of Facebook I would find it extremely disturbing if they were using my data to carry out “health screening.”

##### Usefulness

Some participants were concerned about how effective the software would actually be. Statements were made by referencing the targeted advertisements that are seen on SM because of the use of cookies and search engine histories:

I feel like this already happens for advertising, e.g. I see adverts for online counselling if I share that I've been struggling. I don't always appreciate this though, and it can feel intrusive.

A different subtheme identified within the concern about the usefulness of the software was that the analysis was already being done by friends of users on SM sites:

This is already being done; friends and family already perform this analysis unofficially and take action.

#### Accuracy of Software

Other participants drew attention to worries regarding the software being oversensitive and potentially labeling those who are not suffering with depression with a diagnosis. It was highlighted that some SM users’ posts may contain content that is incorrectly picked up by the machine learning algorithm because of humor or research:

Sometimes people are joking or being sarcastic on Facebook posts, if a person is not mentally well, they need to speak to someone face to face.

In common with many of my friends, I have quite a dark sense of humour and I imagine that my Facebook content might end up flagging concerns incorrectly.

I am always being targeted for things which I am not interested in because I work with vulnerable young people, and my internet activity often reflects this in terms of the articles I read and content I share/groups I join. This doesn’t relate to how I’m feeling, but is research for my work.

## Discussion

### Summary of Key Findings

We recruited a sample of SM users who were demographically broadly representative of the UK population and who mainly used Facebook, Instagram, and Twitter. Participants expressed opinions regarding the feasibility of using SM data to identify depression, and whether, as users of SM, they would agree to this analysis of their online content.

As many of these participants were recruited through mental health charity channels, we had a higher than usual rate of previous depression in our sample (62.2%, 114/183). Only 22.7% (40/176) of the participants who had experienced depressive symptoms believed that low mood would be evident from their posted SM content, and 32% (14/44) of the participants suggested that their SM content is not reflective of their true state of mind when their mood is low. The majority of the participants suggested that they often post less on SM than usual when they are feeling low. These findings are problematic for the approach of analyzing SM content for depression as they suggest that there may be less data available for modeling depression than would be assumed if content was posted at the same level as during positive moods. De Choudhury et al [16] suggest that these changes in SM activity could be used as a feature in a predictive model for depression, in conjunction with the analysis of content, but it is not at all clear how predictive a reduction in activity would be, given that such a reduction could be due to any reason. Inkster et al [33] note that depressed users may stop generating content on SM, so additional data sources, such as text messages and sensor data, could be used to continue monitoring individuals [34].

Participants agreed that they would be worried about their privacy if their SM content was being analyzed for depression, and they did not agree that societal benefits outweighed the risk to their privacy. Privacy concerns were also expressed in the open questions, with participants specifically referencing recent scandals about the use of Facebook data, for example, Cambridge Analytica [35]. Participants were worried that the results of this analysis about mental health could be sold or hacked and may subsequently affect the individual’s insurance premiums or credit ratings, and they endorsed the statement that such analysis could expose a person as having depression. The analysis was also perceived to be intrusive, and 1 participant suggested that it would be “extremely disturbing” for such health screening to be conducted on the SM content, which is viewed as a platform for self-expression. Interestingly, participants rated feeling least comfortable with a human analyzing their content for depression, although they were still largely negative about a computer algorithm conducting the analysis.

We also asked participants whether they would consent to such analysis of their own data. Although a majority of participants were in favor of the idea of this analysis happening in principle, a minority would give consent for their own data to be used in this way and an even smaller minority would be comfortable with it happening without consent. We did not find any differences in the levels of agreement by gender, ethnicity, or history of depression. This lack of support is of interest, given that the profiling of SM users’ demographics and certain content happens without explicit consent already, for targeting advertising within news feeds and across search engines. It suggests that participants may feel qualitatively different about their content being profiled for health status and services compared with advertising for other products. Despite not being in favor of this analysis for their own data, participants could see some benefits in the software being developed, such as identifying and signposting more people to appropriate services and putting current targeted advertising methodologies to a better use.

We have, therefore, identified 3 key issues that weigh with the public when considering the concept of analyzing SM content for signs of depression: (1) that users perceive that the quality of data available may not result in accurate predictions, (2) that they could support the idea of analysis for depression in principle but have key concerns about its safe implementation, and (3) that these concerns center on intrusiveness and risks to privacy. These risks are largely felt to outweigh the benefits of this technology to individuals or society.

### Potential Implications for Services

These findings suggest that SM users hold complex and mixed views on the profiling of content for mental health. They can see some benefits but many have lost trust in certain SM platforms as data custodians, and thus, they regard such analysis as unacceptably intrusive. Although certain mental health charities may be keen to embrace such technologies for advertising services, these findings suggest that the climate may not be right for this approach, and it is possible that charities could lose their clients’ trust if they go down this route. More work is needed to secure a social license for such use of SM users’ data. According to social license theory, which was developed around the ideas of corporate social responsibility by honoring additional safeguards over and above any legal requirements, organizations or corporations may help to engender trust, maintain transparency, and secure societal approval for their activities [36]. Thus, the public looks for a voluntary adherence to the social codes of trustworthy and responsible behavior. When the public is satisfied that the motivations of the organization are trustworthy, their tacit approval can be seen as a “social license” to operate. Previous health data sharing initiatives have collapsed because of failing to secure a social license [37].

### Study Strengths and Limitations

A strength of this study was its mixed methods approach, which created structured quantitative data and also allowed participants to express opinions that were not considered in the questionnaire. Our wide range of questions allowed a comprehensive exploration of the particular aspects of SM analysis for depression that made people uneasy. The open questions revealed strong feelings regarding both the advantages and concerns of the use of this type of software in SM and gave us insights into the reasoning behind some of the responses to structured questions.

However, we relied upon a questionnaire that was created for this study and, thus, has not been validated or replicated in other studies. Some of the questions may need further refinement, and it would be valuable to validate our questionnaire against other similar measures available within the field. Furthermore, despite multiple methods of circulation being used, we secured only a small- to medium-sized sample. Although the demographics of our sample reflect UK averages, they may not represent the typical SM user, where younger age groups tend to dominate. We attempted to increase the number of participants from younger age groups by circulating the questionnaire link through youth-focused sites but had limited success. We purposefully advertised our questionnaire to the types of SM users who might be targeted by mental health service advertisement, and thus, we had a high rate of participants with previous depressive symptoms in our sample. Views of our sample may, therefore, not closely reflect the population in general, although it could be argued that they represent a more informed group of SM users and are, thus, richer in information power [38].

A further limitation would be the timing of the survey. It is likely that the perceptions of risk to privacy and intrusiveness of the use of SM data for secondary purposes were particularly salient in the wake of the Cambridge Analytica scandal, which was revealed in March 2018, when the survey was open. It would be interesting to repeat the survey at a future date to check if the views expressed are stable over time.

### Future Research Directions

Results of this survey suggest a low level of trust in SM platforms to safeguard the users’ privacy and a fear that profiling health status among individuals could lead to harms such as discrimination by insurance or other companies. This may be true for many health conditions, not just depression. Our work could, for example, inform teams that are performing the extraction of information on drug side effects from SM, a field which is currently rapidly expanding [39]. Future work should concentrate on understanding and elaborating the levels of trust in SM platforms and assessing how a social license for reusing SM content for research purposes in health can be achieved. Public sector researchers, such as those at universities, who are conducting this type of work should be mindful of the current climate of distrust and work hard to engage stakeholders in all aspects of their research design, data analysis, and implementation.

### Conclusions

We have shown that the public holds complex views on their SM content being used for targeting advertising for depression services. Although they support the idea in theory, participants in our sample suggested that their main concerns centered on the risks to privacy and considered that the benefits offered by this analysis did not outweigh the privacy risks. Furthermore, a majority of the participants indicated that they would not consent to their data being used for such analysis. This study focused on depression specifically, but such findings may hold across a number of health conditions, especially if they are stigmatized or public health services for them are lacking. Future work in this field should proceed with caution, given users’ current lack of trust in SM platforms, and at a minimum should engage with key stakeholders, such as SM users, at all parts of the research process, to ensure that a social license for research is realized.

## Appendix

Multimedia Appendix 1 Study questionnaire.

## Abbreviations

CMD: common mental disorder
GP: general practitioner
NHS: National Health Service
SM: social media
