# Supplementary material for: Public Opinions on Using Social Media Content to Identify Users With Depression and Target Mental Health Care Advertising: Mixed Methods Survey
Source: JMIR Ment Health. 2019 Nov 13;6(11):e12942. doi: 10.2196/12942 (PMC6881781; doi:10.2196/12942)
Supplement: Multimedia Appendix 1 [file mental_v6i11e12942_app1.pdf]

What do you think about your social media content being analysed for signs of depression?

<u>What is the study about?</u>
Nearly 2 in 3 people with a mental health problem, such as anxiety or depression, do not get diagnosed or treated through NHS services. Many of these people use social media. From people's social media posts, it might be possible to identify who is depressed - and then mental health charities could provide information about their services to these people through adverts on social media sites, such as Facebook.

We are interested in finding out people's opinions as to whether this would be acceptable to social media users.

In this questionnaire there are a range of questions about your use of social media, your privacy preferences in social media, whether you've ever experienced depression or low mood, whether you think social media posts would reflect this low mood, and how you would feel about computer software analysing your social media posts to identify possible depression.

The questionnaire will take roughly 10 minutes to fill in.

<u>Who can take part?</u>
Anyone can take part, ***whether or not they have experienced, or sought medical care for,*** *symptoms of sadness or depression, anxiety, or stress.*
You need to be aged over 16 to take part.

<u>What will I have to do?</u>
You can fill in the online questionnaire, completely anonymously, without giving any identifying details. If you would like to receive a summary of the results, you will need to supply an email address. We will not store your email address with your answers to the questionnaire.

<u>What happens with my answers?</u>
Your answers will be downloaded anonymously and used for statistical analysis.
Where you have provided an email address, this will immediately be removed and stored separately from your answers. Data from the online questionnaire will be stored securely on University of Brighton computer servers. The anonymous database created by this study may be used for several analyses, as we want to find out as much as we can about the relationship between social media activity and mental health.

<u>What happens if I decide that I want to withdraw?</u> Your participation in this study is entirely voluntary and you are free to stop filling in the questionnaire at any time. Once you have

submitted your answers, we will not be able to later remove you from the study, as we will not be able to tell which data belongs to you.

<u>Who has reviewed this study?</u> This study has been reviewed and given favourable ethical opinion by Brighton and Sussex Medical School Research Governance and Ethics Committee (RGEC). If you have a concern about any aspect of this study, you should contact the supervisor Dr Liz Ford who will answer your questions (01273 641974; e.m.ford@bsms.ac.uk). If you have any further questions please contact the researchers: Mr Keegan Curlewis (medical student) [K.Curlewis1@uni.bsms.ac.uk] or Dr Liz Ford (supervisor) 01273 641974; e.m.ford@bsms.ac.uk.

X→ X→

<u>Consent</u>
Please indicate your consent to take part in this study.

○ I have read all the information above and wish to take part in the study

---

Page Break

Q1 What is your age? **You must be older than 16 to participate in this survey.**
Age

▼ 16 ... 99

Q2 What is your gender?

- ◯ Male
- ◯ Female
- ◯ Other

Q3 What is your ethnic background?

- ◯ White British
- ◯ Any other White background
- ◯ White and Black Carribbean
- ◯ White and Black African
- ◯ White and Asian
- ◯ Any other mixed ethnic background
- ◯ Indian
- ◯ Pakistani
- ◯ Bangladeshi
- ◯ Chinese
- ◯ Any other Asian background
- ◯ Black African
- ◯ Black Caribbean
- ◯ Any other Black background
- ◯ Arab
- ◯ Any other ethnic background not stated

Q4 Do you use any of the following social media platforms? Please select as many that apply.

	Yes	No
Facebook	○	○
Twitter	○	○
Tumblr	○	○
Instagram	○	○
Snapchat	○	○
Flickr	○	○
Other - if so, please state what sites.	○	○

Q5 On average, how often do you interact with the social media platforms you chose above? Please select from **only** the social media that you use.

	Many times a day	Once a day	A few times a week	Less than once a week
Facebook	○	○	○	○
Twitter	○	○	○	○
Tumblr	○	○	○	○
Instagram	○	○	○	○
Snapchat	○	○	○	○
Flickr	○	○	○	○
Other	○	○	○	○

Page Break

The following questions will be specifically about social media and mental health.

Q6 Have you ever experienced depressive symptoms long or severe enough you have thought about seeking help?

- Yes
- No

Q7 If you have experienced low mood in the recent past, do you think this would be evident from your online public social media activity?

- Yes
- No

Q8 Is your posted social media content reflective of your true state of mind when you are feeling low?

- Yes
- No

Q9 How much do you tend to post when your mood is very low?

- ◯ More than usual
- ◯ Same as usual
- ◯ Less than usual

Q10 When you are feeling low, do you appreciate getting support from friends on social media?

- ◯ Yes
- ◯ No

Q11 Do you post on social media specifically to seek support for your low mood?

- ◯ Yes
- ◯ No

Q12 Thinking specifically about Facebook:
Please select the type of content you post

	Publicly	To Friends only	To Closed groups	To Open/interest groups	I don't share this
Share articles/pictures/quotes from other sources	○	○	○	○	○
Describe my current life events/share my news	○	○	○	○	○
Describe my state of mind	○	○	○	○	○
Ask for advice or support	○	○	○	○	○
Advertise goods or services/ seek goods or services	○	○	○	○	○
Other - if so, please be specific and tell us the type of content	○	○	○	○	○

X→ X→

Q12b
Do you agree with this statement? "I'm aware of or concerned with the privacy settings when I post on social media".

○ Yes

○ No

Page Break

Social media websites, such as Facebook, are free to use because users' data is used by companies for targeting advertising. It may be possible for this publically available data to be used for targeting information about mental health services to people who need them, such as those who are experiencing low moods.
Thinking specifically about this:

Q13 Would you feel comfortable if you discovered that posts on Facebook were being analysed, in order to target individuals for:

	I would feel very comfortable	I would feel comfortable	I would feel **neither** comfortable **or** uncomfortable	I would feel uncomfortable	I would feel very uncomfortable
Advertising from brands and businesses	○	○	○	○	○
Health care advice, e.g. from the NHS	○	○	○	○	○
Mental health care/advice	○	○	○	○	○
Services offered by mental health charities e.g. Samaritans, Mind or Turning Point	○	○	○	○	○

Q14 It may be possible, using compute programming software, to work out from Facebook content, whether a user is depressed or experiencing low mood in order to provide information about services that may be available. If this technique is shown to work well:

	Agree	Disagree
In general, I support the idea of the use of this software.	○	○
I would give consent for my Facebook content to be analysed for depression.	○	○
I would be comfortable with my Facebook content being analysed for depression **without my explicit consent**	○	○

Q15 How much do you agree with the following statements about analysing Facebook users' content for depression?

	Strongly agree	Agree	Neither agree nor disagree	Disagree	Strongly disagree
It would increase stigmatisation.	○	○	○	○	○
People might end up being "outed" as having depression.	○	○	○	○	○
It would make me feel uneasy.	○	○	○	○	○
I would find this intrusive.	○	○	○	○	○
It would increase people's access to mental health services.	○	○	○	○	○
It could identify people who struggle to seek help for depression in real life.	○	○	○	○	○
I would be worried about my privacy if my Facebook was analysed in this way.	○	○	○	○	○
The benefit to society outweighs the risk to my privacy.	○	○	○	○	○

The benefit to individuals outweighs the risk to my privacy.	○	○	○	○	○
I would feel comfortable if I knew this was happening.	○	○	○	○	○
I would feel comfortable if I knew a human was analysing my Facebook content for depression.	○	○	○	○	○
I would feel comfortable if a computer algorithm (not a human) was analysing my Facebook content for depression.	○	○	○	○	○

Q16 Please tell us what you think would be **beneficial** about analysing Facebook content for depression.

____

____

____

____

____

Q17 What **concerns** would you have about analysing Facebook content for depression?

____

Q18 Any other thoughts or comments?

Q27 Would you like to be added into our prize draw to win a £50 Amazon voucher? If so, please enter your email address below. **Your email address will not be stored with your answers and we will not use it to contact you for any other reason. It will be deleted after the winner is contacted.**

If you have any worries or concerns, or would like further information about mental health services that are available, the following mental health services are accessible:

Your named **GP** is your first and main contact if you need any support or advice.

If you have any questions about this study, please contact **Dr Liz Ford** (01273 641974; e.m.ford@bsms.ac.uk)

Support groups and charities: Samaritans: 116 123 (Open 24 hours a day) Mind: https://www.mind.org.uk/information-support/
0300 123 3393 (9am-6pm, Monday – Friday) Turning Point:
Various services and help can be accessed via their website:

http://www.turning-point.co.uk/mental-health.aspx

End of Block: Default Question Block

Reference to paper

This is a Multimedia Appendix to a full manuscript published in the J Med Internet Res. For full copyright and citation information see http://dx.doi.org/10.2196/jmir.12942.
